# The Treatment of Pancreatitis

**Published:** 1983

**Authors:** N. Setakis

**Affiliations:** Torbay Hospital, Torquay, Devon


Bristol Medico-Chirurgical Journal January/April 1983

### Treatment of Acute Pancreatitis advantages of Glucagon over Propantheline Bromide

**N. Setakis, M.D., A. M. N. Gardner, M.Ch. and D. Fox, M.App.Sc.**
Torbay Hospital, Torquay, Devon

### INTRODUCTION

Published reports suggest a considerable geographical variation in the pathogenesis of acute pancreatitis (Table 1). This may account for the contradictory findings regarding the effectiveness of the commonly used therapeutic agents Propantheline Bromide (Pro-Banthine), Aprotinin and Glucagon. In most studies, the majority of cases have been associated with biliary disease, an undetermined proportion having a stone in the ampulla of vater obstructing the common opening of the bile and pancreatic ducts so enabling bile to reflux into the pancreas (Opies syndrome). The study here reported was prompted by our own experience of the remarkable effectiveness of Glucagon not only in reducing mortality and morbidity of our patients, but also in controlling pain where it has proved as effective as Pethidine in the acute stages of the disease.

Our study is retrospective but allocation to treatment groups was random and treatment groups were statistically comparable in respect of age, sex and serum amylase levels (Tables 2 and 3).

**Table 1**

**Reported Etiological Factors in Acute Pancreatitis**

| | *Biliary disease (%)* | *Alcohol (%)* | *Idiopathic (%)* |
|---|---|---|---|
| Gardner (Torbay) | 84 | 3 | 13 |
| Gillespie (Edinburgh) | 34 | 23 | 38 |
| M.R.C. Trial (Multicentre) | 55 | 9 | 32 |
| Trapnell (Bristol) | 55 | 1 | 37 |
| Ranson (U.S.A.) | 14 | 74 | 12 |

*Address for correspondence:* A. M. N. Gardner, M.Ch., Consultant Surgeon, Torbay Hospital, Torquay TQ2 7AA, Devon.

**Table 2**

**Sample distribution for Serum Amylase**

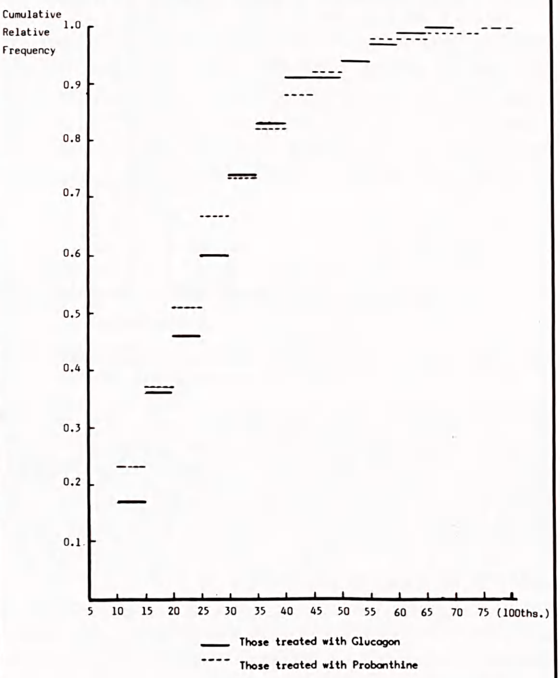

### MATERIALS AND METHODS

One hundred and sixty patients admitted as emergencies with acute pancreatitis to Torbay Hospital during the past nine years were studied. Diagnostic criteria were:

(1) History and clinical signs comparable with acute pancreatitis.

(2) Serum amylase raised to more than twice the maximum normal limit of 300 u/litre Int. Units.

Allocation to treatment groups was random since treatment depended on to which of three surgical services on emergency rota the patients were admitted. On one surgical service, the policy was to

**Table 3**

**Distribution of Serum Amylase Content on Successive Days for Both Groups of Patients**

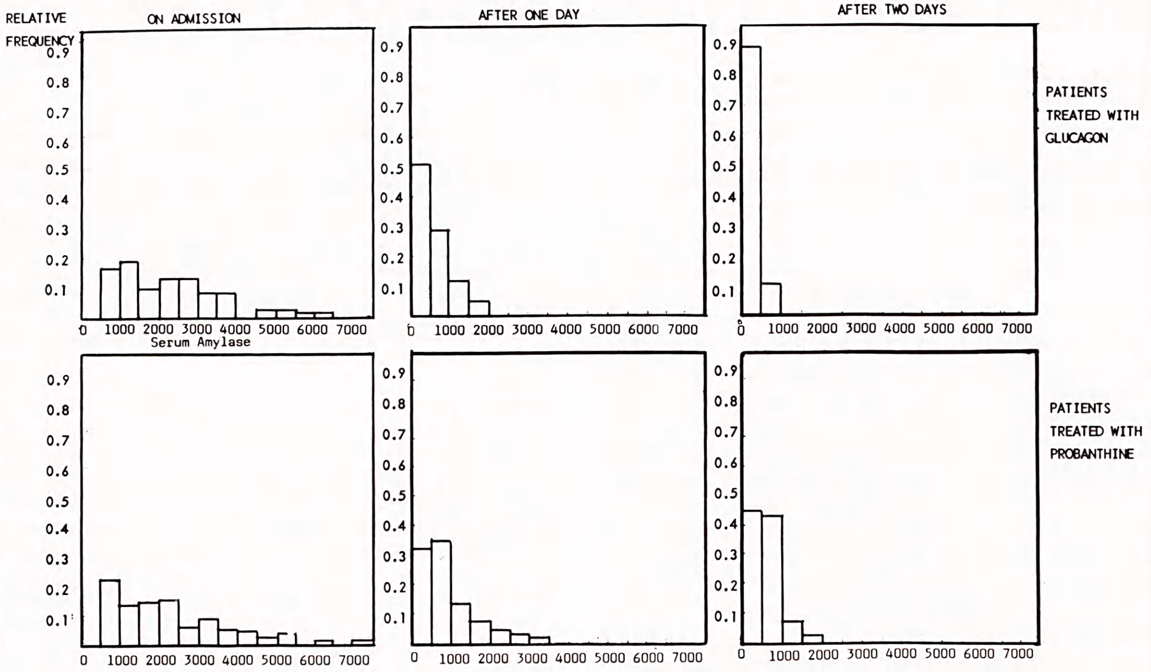

give Glucagon 1 mg six hourly. On the second service, Propantheline 15 mg was given six hourly and on the third, the policy changed from Propantheline to Glucagon during the period of study. In all cases, gastric aspiration and i.v. fluids were given during the acute stage, together with Ampicillin 250 mg to 500 mg six hourly to prevent infection. Excluded from the study were a small number of patients who had developed the disease after biliary surgery and a few who had inadvertently received both Glucagon and Propantheline and a few whose records were inadequate. Statistical study in respect of (sex, age and) severity of disease as judged by amylase levels confirmed the comparability of the two groups (Tables 2 and 3).

Results of treatment were judged by

(a) mortality,
(b) length of stay in hospital,
(c) rate of fall of serum amylase,
(d) amounts of Pethidine required to control pain.

### RESULTS

In the Glucagon group, three of the seventy patients (4%) died, none from pancreatitis, whereas in the Propantheline group, nine of the ninety died (10%), five of them from uncontrolled pancreatitis. Table 4 shows the cause of deaths in the two groups. In

**Table 4**

**Deaths in Acute Pancreatitis in Relation to Treatment**

| CAUSE OF DEATH | GLUCAGON 70 patients | PROBANTHINE 90 patients |
|---|---|---|
| Acute Pancreatitis | 0 | 5 ♀ aet 31 |
| | | ♂ aet 67 |
| | | ♀ aet 70 |
| | | ♀ aet 72 |
| | | ♀ aet 78 |
| Pneumonia | 2 ♂ aet 70 | 3 ♂ aet 69 |
| | ♂ aet 91 | ♀ aet 73 |
| | | ♀ aet 79 |
| Myocardial Infarction | 1 ♀ aet 80 | |
| Pulmonary Embolism | | 1 ♀ aet 56 |
| Totals | 3 = 4% | 9 = 10% |

addition, one patient in the Propantheline group developed a pseduo-pancreatic cyst. The mean length of hosptial stay was ten days with Glucagon and fourteen days with Propantheline. The more rapid recovery with Glucagon was confirmed statistically by a more rapid fall in serum amylase levels. Control of pain in both groups was with Pethidine because unlike other opiates, it is thought not to cause spasm of the sphincter of Oddi. An average of only three doses of Pethidine were given to the Glucagon patients, whereas in the Propantheline group, it was required four hourly at least for 48 hours and sometimes as long as ten days. In the latter part of the study, it was realized that Glucagon was usually as effective as Pethidine in controlling pain and in some cases, no Pethidine at all was given. If pain recurred, an extra dose of Glucagon usually gave relief.

### DISCUSSION

In the treatment of acute pancreatitis, all are agreed on the paramount importance of maintaining fluid and electrolyte balance, but opinions vary in relation to other treatment. On the one hand, some advise no further therapy whereas others, particularly in stone cases, operate to drain the area or to explore the ducts. Protagonists of the latter policy are encouraged by the frequent finding of stones impacted in the ampulla of vater (Opies syndrome). Acosta et al[5] discovered calculi in 33 out of his 45 causes so explored.

Majority opinion favours a conservative approach in the acute disease using anti-cholinergic drugs (Propantheline), anti-trypsin (Aprotinin) or Glucagon. The relative value of these treatments is not decided and our own experience is at variance with that of the M.R.C. Multicentre study[2] in that we found Glucagon to be effective not only in reducing mortality and morbidity but also almost as effective as Pethidine in relieving pain. Our clinical material was not, however, statistically comparable with that of the M. R. C. trial which included a greater proportion of alcohol related and idiopathic cases. Aprotinin should theoretically be helpful and is reported to be beneficial providing treatment is started within 24 hours. Few of our cases have presented within this time and in the past perhaps, because of this, we found response to this treatment to be much less dramatic than that subsequently seen with Glucagon. Propantheline, likewise, should be effective since experimentally pancreatic flow and enzyme secretion are reduced. Unfortunately in the dosage required, the complications of Ileus, urinary retention, tachycardia and visual disturbances are distressing and even dangerous in the ill patient.

The usefulness of Glucagon in pancreatitis was first reported by Knight et al[7] whose findings we have here confirmed. It suppresses both exocrine pancreatic and gastric secretions,[8] reduces gastrointestinal motility and our own studies show that in addition it relaxes the sphincter of Oddi and counteracts the spasmodic effect of opiates. It is this dilatory action that is likely to be responsible for its usual, almost instantaneous action of relieving pain in acute pancreatitis. A possible additional advantage is its inotropic effect[9,10] in dangerously ill patients. In the recent M. R. C. Multicentre trial, comparing Glucagon and Aprotinin in acute pancreatitis,[2] neither treatment was found to diminish the risk of death. However, no direct comparison can be made with our own series because of differences in aetiology, sex, selection criteria and subsequent treatment. In particular, the wide geographic variation in the nature of the disease makes it difficult to compare the various treatments. Possible reasons for our own exceptionally low mortality rate is the routine use of antibiotics (Ampicillin)—not used routinely in some studies, and the fact that almost all our cases were associated with biliary disease, rather than alcohol.

### SUMMARY

In an area where acute pancreatitis is common and usually associated with biliary disease, a retrospective study of statistically comparable groups of patients has clearly shown the therapeutic superiority of Glucagon over Propantheline. Recovery was more rapid and amylase levels fell more quickly. Mortality with Glucagon treatment was 3/70 (4%) and with Propantheline 9/90 (10%). Length of stay in hospital was shorter and analgesic requirements greatly reduced.

### ACKNOWLEDGEMENTS

We grateful to Mr H. P. Guerrier, Mr P. J. W. Monks and Mr J. W. S. Rickett for permission to use their cases in this study.

### BIBLIOGRAPHY

1. Gillespie, W. J. (1973) Observations on acute pancreatitis. *Br.J.Surg.* **60**, 63–65.
2. M. R. C. MULTICENTRE TRIAL OF GLUCAGON AND APROTININ (1977) Death from acute pancreatitis. *Lancet* **24**, 632–635.
3. TRAPNELL, J. E. (1966) Natural history and prognosis of acute pancreatitis. *Ann.R.Coll.Surg.Eng.* **38**, 265–287.
4. RANSON, J. H. C. (1974) Prognostic signs and the role of operative management in acute pancreatitis. *S.G.O.* **139**, 69–73.

5. ACOSTA, J. M., ROSSI, R., GALLI, O. M. R., PELLEGRIN, C. A. and SKINNER, D. (1978) Early surgery for acute gallstone pancreatitis, evaluation of a systemic approach. *Surgery* **83**, 367–369.
6. DREILING, D. A., LEICHTLING J. J. and GREENSTEIN, A. J. (1976) Trasylol revisited: the value of proteolytic inhibitors in the therapy of pancreatitis. *The Mt.Sinai J.Med.* **43**, 409–414.
7. KNIGHT, M. J., CONDON, J. R. and SMITH, R. (1971) Possible use of glucagon in the treatment of pancreatitis. *Br.Med.J.* **2**, 440–442.
8. DYCK, W. P., TEXTER, E. C., LASATER, J. H. and HIGHTOWER, N. C. (1970) Influence of glucagon on pancreatic exocrine secretion in man. *Gastroenterology* **58**, 532–539.
9. CONDON, J. R., KNIGHT, M. and DAY J. L. (1973) Glucagon therapy in acute pancreatitis *Br.J.Surg.* **60**, 505–509.
10. DOTERALL, G. and KOCK, N. G. The effect of glucagon on intestinal motility in man. *Gastroenterology* **45**, 364–367.

